# Association of Insulin Resistance With Cardiovascular Risk Factors and Sleep Complaints: A 10-Year Follow-Up

**DOI:** 10.3389/fpubh.2022.848284

**Published:** 2022-05-16

**Authors:** Aurelija Podlipskyte, Nijole Kazukauskiene, Giedrius Varoneckas, Narseta Mickuviene

**Affiliations:** Laboratory of Behavioral Medicine, Neuroscience Institute, Lithuanian University of Health Sciences, Palanga, Lithuania

**Keywords:** insulin resistance, sleep complaints, cardiovascular risk, metabolic syndrome, aging

## Abstract

The aim of the study was to investigate the association of insulin resistance (IR) with cardiovascular risk factors and sleep complaints among citizens of Palanga over a 10-year follow-up period. This epidemiological longitudinal cohort study was performed with 835 subjects.

**Methods:**

All participants were evaluated for sociodemographic, clinical and cardiovascular risk factors, behavioral factors, self-perceived health and biochemical analysis. IR was evaluated using the homeostasis model assessment of IR (HOMA-IR).

**Results:**

All study participants were stratified into two groups, without IR (HOMA-IR ≤ 2.7) and with IR (HOMA-IR > 2.7). The analysis of parameters between the two study groups showed statistically significant relationships between IR, cardiovascular risk factors and sleep complaints within the 10-year period. After adjusting for a 10-year period, sex, age, body mass index, physical activity, education, systolic and diastolic blood pressures, presence of disease, total cholesterol, triglyceride levels, metabolic syndrome (MetS) and diabetes mellitus (DM), IR was statistically significantly more frequent in subjects with increased sleep latency [odds ratio (OR) 1.37, 95% CI 1.01–1.93; *p* = 0.043], snoring frequency (OR 1.37, 95% CI 1.05–1.79; *p* = 0.020) and very loud snoring (OR 1.34, 95% CI 1.04–1.74, *p* = 0.026).

**Conclusions:**

The incidence of obesity, MetS, DM, elevated fasting glucose level, triglyceridemia and sleep complaints became more frequent after a 10-year period in subjects with IR. Over a 10-year period, IR was significantly associated with an increase in sleep complaints: sleep latency reflecting difficulty to fall asleep, snoring and very loud snoring.

## Introduction

Insulin resistance (IR) is characterized by an impaired glucose metabolic response to a given amount of insulin and is considered to contribute to the development of diabetes mellitus (DM) and to be a major risk factor for cardiovascular diseases (1). Cardiovascular disease is the most prevalent cause of morbidity and mortality in diabetic populations (2). A systematic review has confirmed that IR, as measured by the homeostasis model assessment of IR (HOMA-IR) is independently associated with greater risk of incident cardiovascular disease and all-cause mortality in non-diabetic adults (3). The assessment of IR indicators in everyday clinical practice is based on fasting glucose and insulin concentrations. The HOMA-IR is one of the most frequently used indirect indicators for characterizing this condition in the “steady-state” (4, 5).

A group of cardiovascular and metabolic abnormalities, such as abdominal obesity, hypertension, dyslipidaemia, atherosclerosis, and IR predispose individuals to cardiovascular disease and type 2 DM (6, 7). Compensatory hyperinsulinemia, like other components of metabolic syndrome (MetS), is closely associated with an increased risk of cardiovascular disease (8, 9). The main components of IR are hyperinsulinemia, hypertriglyceridemia, dyslipidaemia, hypertension and obesity (10). Therefore, IR is a substantial cardiovascular risk factor in healthy people. Normalizing IR reduced cardiovascular risk by approximately 55% in subjects with this condition. IR was the most significant single risk factor for coronary artery disease in young people, accounting for nearly half of all myocardial infarctions (11). It is generally present for many years before the diagnosis of DM and causes subclinical vascular damage. Normoglycemic individuals with IR are already at an elevated risk of cardiovascular disease years before the onset of clinical DM (12).

Cross-sectional studies have shown that sleep disordered breathing impairs glucose tolerance and/or insulin sensitivity, as measured by HOMA-IR, even after adjusting for body mass index (BMI) and in non-diabetic patients (13). Furthermore, the prevalence of IR, glucose intolerance, type 2 DM and cardiovascular diseases is reported to be 20–67% higher in subjects with sleep disorders than in controls (14). Moreover, sleep-disordered breathing is also an independent risk factor for coronary heart disease, heart failure and stroke (15). Short and long sleep times are associated with lower insulin sensitivity, suggesting that sleep plays an important role in IR and may be associated with DM development (16). Short sleep time is independently associated with IR in women, even after adjusting for body fat and other potentially dependent factors (17). From the otherperspective, DM has a negative impact on the development of cardiovascular diseases (18). According to epidemiological research, sleep duration is associated with unfavorable cardiovascular outcomes, such as coronary artery disease, stroke, and cardiovascular death (19, 20), as well as metabolic problems (21). In a study by Vargas et al. (22), poor sleep quality in women with severe obesity showed a negative association with body fat, metabolic outcomes and fitness. As a result, poor sleep quality is a significant factor that can exacerbate the health of women with severe obesity.

To date, correlations between IR and the risk of sleep disorders and cardiovascular diseases during aging have received little attention in the medical literature; therefore, the topic remains controversial, is not well understood and needs further investigation. In order to understand the contribution of sleep as a whole to public health, it is necessary to integrate the concepts of sleep quality and duration in order to assess their combined and independent influence on health outcomes. This raises the question of how the modifications of cardiovascular risk factors and sleep complains evolve according to the presence of IR during aging. Thus, we aimed to investigate the association of IR with cardiovascular risk factors and sleep complaints over a 10-year follow-up period.

## Materials and Methods

### Study Participants

**Figure 1:**
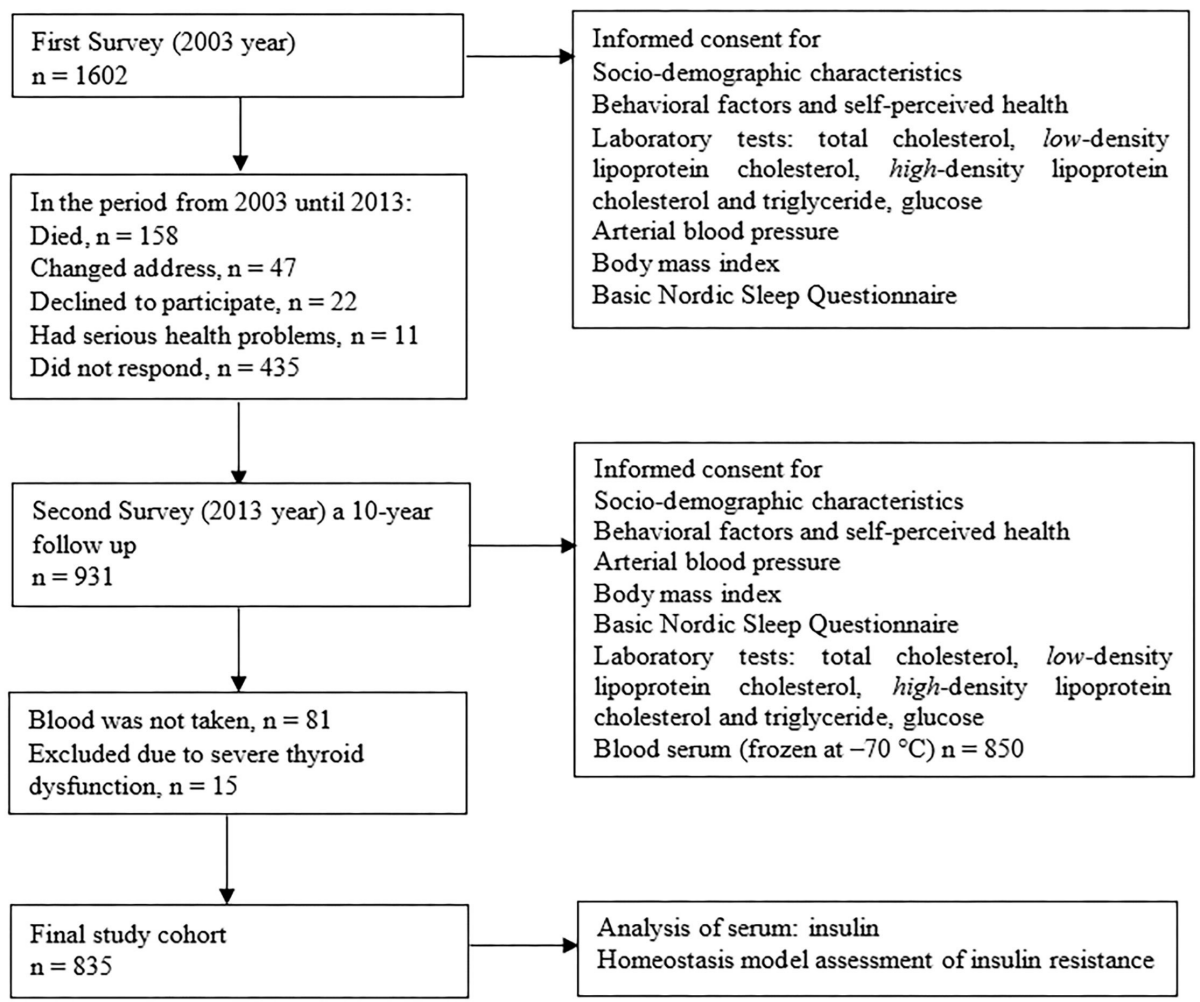
Study flow chart.

A sample of 2,500 citizens of Palanga aged 35–74 years was drawn from the National Population Register in 2002. The citizens of Palanga were chosen for the investigation, because there was close community with minor migration reflecting the population of the western part of Lithuania. The optimal size of the sample, ensuring representativeness of the population of Palanga aged 35–74 years, was calculated as 1,630 ± 33 subjects. From the sample of 2,500 citizens, 160 were not invited to participate in the study because they were not found at the given address. In total, 1602 persons (600 males and 1,002 females) participated in the survey in 2003. The response rate for the first survey (2003) was calculated as follows: (1,602/2,340) ×100 = 68.5%. In the period from 2003 to 2013, 158 of the participants in the first survey in 2003 died (9.9%) and 47 (2.9%) had changed their address. When contacted, 20 (1.2%) declined to participate, 11 (0.7%) could not participate as a result of serious health problems, and 435 (27.2%) did not respond to multiple invitations sent to them by post. During the second survey, data from 931 people, 322 males and 609 females, aged 45–84 years, were collected. The first and second surveys were approved by the Bioethics Committee of Lithuania (Protocol code BE-2-25, approved 14 June 2012). Informed consent was obtained from all participants during both surveys. However, blood samples were collected from only 850 subjects and 15 (1.8%) subjects, whose blood tests showed severe thyroid dysfunction, were excluded from the analysis. The final longitudinal study cohort consisted of 835 subjects: 300 (35.9%) men and 535 (64.1%) women (Figure 1). The mean age of the study group was 63.5 ± 10.3 years. The methods are originally described elsewhere (23).

### Study Procedure

All study participants were evaluated according to socio-demographic characteristics (i.e., age, gender, height, weight, education, marital status, type of job), behavioral factors and self-perceived health using a questionnaire. Fasting blood samples were taken from all participants and biochemical tests were performed to assess glucose, insulin, total cholesterol, low-density lipoprotein (LDL) cholesterol, high-density lipoprotein (HDL) cholesterol and triglyceride levels. IR was calculated according to the HOMA-IR. MetS was defined according to the Adult Treatment Panel III (ATPIII) (24) and International Diabetes Federation (IDF) (25) criteria. Under the ATPIII criteria, MetS was defined as the presence of three or more of the following risk factors: abdominal obesity (waist circumference ≥102 cm [men] or ≥88 cm [women]), triglyceride ≥1.7 mmol/L (150 mg/dL), HDL-cholesterol <1.03 mmol/L (40 mg/dL) in men and <1.30 mmol/L (50 mg/dL) in woman, fasting glucose ≥6.1 mmol/L (110 mg/dL) and systolic blood pressure or >130 mm Hg or diastolic >85 diastolic mm Hg. MetS was identified when three or more of the five components mentioned above were present.

### Measures

#### Questionnaire on General Data, Behavioral Factors and Self-Perceived Health

The questionnaire on general data (26) was used to collect the information about the marital status, education, employment and income of respondents. The questionnaire on behavioral factors (26) contained questions about smoking, alcohol consumption, and physical activity during the last year. The questionnaire on self-perceived health (26) contained questions about complaints and diagnosed diseases, medicines used during the last year, frequency of stress events, and visits to any doctor.

#### Objective Investigation

Arterial blood pressure (mmHg) was measured twice using a quicksilver sphygmomanometer (Riester 660/306, DIPLOMAT Presameter, Germany) from the right arm while the person was sitting, with the precision of 2 mm referring to the methodological recommendations (27). An average of two measurements was used for the analysis. The participants were classified as hypertensive if their systolic blood pressure was ≥140 mmHg and/or diastolic arterial blood pressure was ≥90 mmHg, or if they had received antihypertensive drug treatment in the last 2 weeks.

Body height was measured in stocking feet (without shoes) using a medical height rod. Body weight was measured without shoes using a medical scale (SECA 778, SECA Corporation, Hamburg, Germany). BMI was calculated according to the following formula: BMI = body mass (kg)/height^2^ (m) using the data from the height and weight measurement. Patients were diagnosed as being overweight when their BMI was 25.0–29.9 kg/m^2^, and with obesity when their BMI was 30.0 kg/m^2^over. Waist circumference was measured during the physical examination. Weight and height were measured with participants standing, without shoes or heavy outer garments. Waist circumference was measured in the erect position at the midpoint between the lowest rib and the superior border of the iliac crest.

#### Basic Nordic Sleep Questionnaire

The Basic Nordic Sleep Questionnaire (28) was used to assess the frequency of sleep complaints over a 3-month period in both the first and second surveys. The self-administered questionnaire included questions about problems falling asleep, awakenings during the night and too early in the morning, self-rated sleep quality, excessive sleepiness during the daytime, the effect of sleep on the ability to work, napping during the daytime, the regular use of sleeping pills and the use of alcohol to initiate sleep. The criteria for sleep complain was a repetition of sleep disturbance symptom(s) 3–5 times per week for the last 3 months.

#### Laboratory Tests

Blood serum analyses were performed in the biochemical testing laboratory. The participants were warned in advance to have no intake of food for at least 12 h before the tests. Blood was taken from the elbow vein directly into a vacuum blood collection systems, while the person was sitting (7 ml). Blood samples were analyzed for concentrations of glucose (mmol/L, norm 4.1–5.9), total cholesterol (mmol/L, norm 2.6–5.2), LDL cholesterol (mmol/L, norm 2.6–3.4), HDL cholesterol (mmol/L, men's norm 0.9–1.7), women's, norm 0.9–2.0), and triglycerides (mmol/L, norm 0.5–2.3) in both 2003 and 2013. The blood obtained in 2013 was centrifuged and the serum was frozen at −70° C. Serum samples of insulin were analyzed in a single batch after completion of this study in 2013 (*n* = 850, mU/L, norm 3.0–25.0). IR was calculated according to the HOMA-IR formula: HOMA-IR = [fasting plasma insulin (μIU/ml)] × [fasting plasma glucose (mmol/l)]/22.5); normal ≤ 2.7 (29, 30).

#### Statistical Analysis

The clinical and the sociodemographic characteristics were reported using frequencies and percentages for the categorical variables, and means and standard deviations for the continuous variables, and medians (25–75th percentiles) for variables with non-normal distribution. Variable distribution was assessed visually and using the Kolmogorov-Smirnov test. The data characteristics were compared between the groups without and with IR were using Fisher's χ^2^ test, the parametric two-tailed Student's *t*-test test or the non-parametric Mann-Whitney U test. A logistic regression analysis using an enter method was used to investigate whether IR is related to different sleep complaints (Model 1) and adjusted for the 10-year period, sex, age, body mass index, physical activity, education, systolic and diastolic blood pressures, presence of disease, total cholesterol, triglycerides, MetS, and DM (Model 2), (**Table 3**). A logistic regression analysis using an enter method was used to investigate whether IR was related to cardiovascular risk factors (Model 1) and adjusted for the 10-year period, sex, age, and education (Model 2), (**Table 4**). The dependent variables in all models were all sleep complaints and cardiovascular risk factors in 2013. Statistical analyses were performed using the Statistical Package for the Social Sciences v.22 (SPSS, Chicago, IL). The threshold of significance was set at *p* < 0.05.

## Results

### Comparison of Baseline Sociodemographic Characteristics in the Survey in 2003 and 2013 According to the Presence of IR

Table 1 lists the sociodemographic characteristics of all participants stratified into groups without IR (HOMA-IR ≤ 2.7) and with IR (HOMA-IR > 2.7) in 2013. As demonstrated in Table 1, the sociodemographic characteristics statistically significantly changed in both groups after the 10-year follow-up; however, the directions of changes of individual parameters in the IR group and the group without IR were different. Marital status significantly changed in both groups after the 10-year period. A decrease in married status and an increase in living alone was statistically significant in both groups. The group without IR, was characterized by more people with higher education at both timepoints. The employment rate statistically significantly decreased in both groups, but with a larger decrease in the IR group.

**Table 1:** The sociodemographic and cardiovascular risk factors characteristics of subjects' in the survey in 2003 and 2013 according to the presence of insulin resistance.

**Baseline characteristics**	**2003**		***p*-value**	**Cohen's *d*** **Cramer's *V***	**2013**		***p*-value**	**Cohen's *d*** **Cramer's *V***	**2003:2013**	**2003:2013**
	**Without IR** **group**	**With IR** **group**			**Without IR** **group**	**With IR** **group**			**Without IR** **group**	**With IR** **group**
	***n =* 557**	***n =* 278**			***n =* 557**	***n =* 278**				
Age, years; mean ± SD	53.1 ± 10.6	54.9 ± 9.9	**0.016**	0.176	63.0 ± 10.5	64.8 ± 9.7	**0.015**	0.178	**<0.001**	**<0.001**
Gender; *n* (%)			**0.003**	0.098			**0.003**	0.098	–	–
Male	181 (32.5)	119 (42.8)			181 (32.5)	119 (42.8)				
Female	376 (67.5)	159 (57.2)			376 (67.5)	159 (57.2)				
Marital status; *n* (%)			0.932	0.005			0.533	0.024	**0.004**	**0.007**
Married	420 (75.4)	211 (75.9)			376 (67.5)	181 (65.1)				
Alone	137 (24.6)	67 (24.1)			181 (32.5)	97 (34.9)				
Education; *n* (%)			**0.001**	0.235			**0.001**	0.254	0.287	0.839
Less than higher	369 (66.2)	217 (78.1)			351 (63.0)	214 (77.0)				
Higher	188 (33.8)	61 (21.9)			206 (37.0)	64 (23.0)				
Employment; *n* (%)			0.281	0.040			0.142	0.052	**<0.001**	**<0.001**
Employed	369 (66.2)	173 (62.2)			277 (49.7)	123 (44.2)				
No employed	188 (33.8)	105 (38.8)			280 (50.3)	155 (55.8)				
Systolic blood pressure, mm Hg; mean ± SD	120.3 ± 17.0	127.2 ± 16.0	**<0.001**	0.418	128.3 ± 12.9	132.2 ± 12.5	**<0.001**	0.307	**<0.001**	**<0.001**
Diastolic blood pressure, mm Hg; mean ± SD	81.9 ± 7.2	86.7 ± 9.4	**<0.001**	0.573	74.0 ± 7.2	75.7 ± 7.4	**0.002**	0.233	**<0.001**	**<0.001**
Presence of Disease; n (%)			**<0.001**	0.216			**0.001**	0.136	**<0.001**	**<0.001**
Without AH and CAD	422 (75.9)	156 (56.1)	**<0.001**		122 (21.9)	37 (13.3)	**0.003**		**<0.001**	**<0.001**
AH	39 (7.0)	27 (9.7)	0.172		256 (46.0)	142 (51.1)	0.163		**<0.001**	**<0.001**
CAD	65 (11.7)	27 (9.7)	0.395		35 (6.3)	8 (2.9)	**0.034**		**0.002**	**0.001**
With AH and CAD	30 (5.4)	54 (14.7)	**<0.001**		144 (25.9)	91 (32.9)	**0.038**		**<0.001**	**<0.001**
BMI, kg/m^2^; mean ± SD	26.0 ± 4.3	30.0 ± 4.5	**<0.001**	0.909	26.6 ± 4.1	31.0 ± 4.7	**0.002**	0.998	**0.013**	**0.005**
BMI ≥ 30; *n* (%)	86 (15.4)	133 (47.8)	**<0.001**	0.347	99 (17.8)	162 (58.3)	**<0.001**	0.412	0.334	**0.017**
Low physical activity; *n* (%)	350 (62.8)	185 (66.5)	0.293	0.035	145 (26.9)	94 (35.2)	**0.015**	0.086	**<0.001**	**<0.001**
Total cholesterol, mmol/L; median (IQR)	5.7 (4.9–6.5)	5.8 (5.2–6.8)	**0.005**	0.207	5.9 (5.2–6.6)	5.9 (4.9–6.8)	0.803	0.018	**0.001**	0.806
LDL, mmol/L; median (IQR)	3.5(2.8–4.3)	3.6 (3.1–4.4)	**<0.001**	0.523	3.7 (3.1–4.3)	3.8 (2.9–4.6)	0.229	0.086	**0.001**	0.621
HDL men's, mmol/L; median (IQR)	1.3 (1.1–1.6)	1.2 (1.0–1.5)	**0.013**	0.525	1.5 (1.2–1.8)	1.2 (1.1–1.5)	**<0.001**	0.555	**<0.001**	0.230
HDL women's, mmol/L; median (IQR)	1.5 (1.3–1.9)	1.4 (1.1–1.6)	**<0.001**	0.337	1.8 (1.5–2.1)	1.5 (1.3–1.8)	**<0.001**	0.516	**<0.001**	**<0.001**
Triglyceride, mmol/l; median (IQR)	1.3 (1.0–2.3)	1.7 (1.2–2.3)	**<0.001**	0.213	1.1 (0.8–1.4)	1.5 (1.1–2.0)	**<0.001**	0.702	**<0.001**	**<0.001**
With metabolic syndrome; *n* (%)	90 (16.2)	139 (50.0)	**<0.001**	0.357	61 (11.0)	143 (51.4)	**<0.001**	0.444	**0.014**	0.799
Smoking regular; *n* (%)	198 (35.5)	112 (40.3)	0.182	0.064	85 (15.3)	38 (13.7)	0.541	0.021	**<0.001**	**<0.001**
Diabetes mellitus; *n* (%)	6 (1.1)	23 (8.3)	**<0.001**	0.185	17 (3.1)	45 (16.2)	**<0.001**	0.236	**0.033**	**0.006**
Fasting glucose, mmol/L; mean ± SD	5.5 ± 0.9	6.1 ± 1.6	**<0.001**	0.462	5.0 ± 0.5	6.1 ± 1.7	**<0.001**	0.878	**<0.001**	0.886
Fasting glucose ≥6.1; *n* (%)	115 (20.7)	108 (39.0)	**<0.001**	0.195	8 (1.4)	87 (31.3)	**<0.001**	0.443	**<0.001**	0.334

*IR, insulin resistance; AH, arterial hypertension; CAD, coronary artery disease; BMI, body mass index; LDL, low-density lipoprotein cholesterol; HDL, high-density lipoprotein cholesterol; p-value of probability for comparison between groups (p <0.05 in bold); SD, standard deviation; data presented as n (%), mean ± SD and median (IQR)-−25–75 percentiles*.

### Comparison of Cardiovascular Risk Factors in the Survey in 2003 and 2013 According to the Presence of IR

An increase of number of participants with high BMI (≥30) was seen in the IR group compared with the without IR group (Table 1). However, after the 10-year period, the number of people with DM increased in both groups, while the number of people with MetS and fasting glucose ≥6.1 (mmol/L) was decreased. The comparison of cardiovascular risk factors before and after the 10-year period showed some statistically significant differences between the two groups. Systolic and diastolic arterial blood pressure was greater in subjects with IR at both timepoints. However, diastolic arterial blood pressure decreased in both groups after 10 years. There were also significantly fewer smokers in both groups after 10 years. The number of people with cardiovascular disease statistically significantly increased after the 10-year period in both groups; however, co-morbid diseases such as arterial hypertension and coronary artery disease were more frequent in the IR group. After the 10-year period, total cholesterol and LDL levels statistically significantly increased in the group without IR, reaching a level similar to the IR group' while in the IR group, total cholesterol and LDL levels remained the same. In men, HDL level increased in both groups; however statistically significant changes were seen only in the group without IR. In women, there was an opposite change in HDL, with the decrease after the 10-year period being statistically significant in both groups. Triglyceride levels also statistically significantly decreased after the 10-year period in both groups.

### Comparison of Sleep Complaints in the Survey in 2003 and 2013 According to the Presence of IR

Analysis of sleep complaints showed statistically significant changes in the IR group after the 10-years period (Table 2). In 2013, the participants in this group were more likely to complain of difficulty falling asleep and early waking in the morning (*p* = 0.003), resulting in more frequent severe drowsiness in the morning (*p* < 0.001) and during the day (*p* = 0.002). Daytime sleepiness was also more frequently observed among people with IR. They were also more likely to use sleeping pills (*p* = 0.034) and, unsurprisingly, were more likely to use alcohol to improve sleep (*p* = 0.012).

**Table 2:** Prevalence of sleep complaints and sleep parameters in the survey in 2003 and 2013 according to the presence of insulin resistance.

**Sleep complaints**	**2003**		***p*-value**	**Cramer's V**	**2013**		***p*-value**	**Cramer's V**	**2003:2013**	
	**Without IR** **group**	**With IR** **group**			**Without IR** **group**	**With IR** **group**			**Without IR** **group**	**With IR** **group**
	***n =* 557**	***n =* 278**			***n =* 557**	***n =* 278**				
		***n*** **(%)**								
Difficulties falling asleep ≥ 3 nights per week	56 (10.1)	31 (11.2)	0.633	0.017	87 (15.7)	49 (17.8)	0.486	0.026	**0.005**	**0.031**
Sleep Latency >30 min.	53 (9.5)	40 (14.4)	**0.047**	0.073	71 (12.7)	43 (15.5)	0.286	0.037	**0.086**	0.722
Difficulties maintaining sleep ≥ 3 nights per week	223 (40.0)	131 (47.1)	*0.054*	0.068	276 (49.4)	139 (50.0)	0.941	0.004	**0.001**	0.504
Awakenings ≥ 3 times per night	80 (14.4)	38 (13.7)	0.883	0.009	80 (14.4)	53 (19.1)	*0.088*	0.061	1.00	*0.087*
Awakenings too early in the morning ≥ 3 times per week	71 (12.7)	35 (12.6)	1.00	0.002	92 (16.5)	68 (24.5)	**0.007**	0.095	*0.075*	**0.003**
Poor self-perceived sleep	45 (8.1)	28 (10.1)	0.363	0.033	54 (9.7)	33 (11.9)	0.338	0.034	0.344	0.500
Excessive sleepiness in the morning ≥ 3 times per week	65 (11.7)	25 (9.0)	0.287	0.041	97 (17.4)	51 (18.3)	0.773	0.011	**0.007**	**0.001**
Excessive sleepiness in the day ≥ 3 days per week	50 (9.0)	19 (6.8)	0.315	0.037	93 (16.7)	42 (15.1)	0.618	0.020	**<0.001**	**0.002**
Inability to work due to disturbed sleep ≥ 3 days per week	37(6.6)	18 (6.5)	1.00	0.003	24 (4.3)	10 (3.6)	0.713	0.017	*0.087*	0.122
Sleeping time ≤ 6 h	437 (78.5)	217 (78.1)	0.458	0.031	448 (80.4)	206 (74.1)	0.320	0.041	0.415	0.300
Snoring ≥ 3 nights per week	131 (23.6)	96 (34.5)	**<0.001**	0.115	94 (17.2)	83 (30.4)	**<0.001**	0.150	**0.005**	0.238
Very loud Snoring	24 (4.3)	24 (8.6)	**0.012**	0.088	43 (7.7)	28 (10.1)	0.292	0.040	**0.017**	0.662
Breathing pauses ≥ 3 nights per week	10 (1.8)	12 (4.3)	**0.032**	0.074	25 (4.8)	19 (7.4)	0.140	0.049	**0.010**	0.267
Napping during the daytime ≥ 3 days per week	55 (10.6)	25 (9.0)	0.542	0.025	60 (10.8)	41 (14.7)	0.115	0.057	0.623	**0.037**
Regular use of sleeping pills ≥ 3 days per week	18 (3.2)	11 (4.0)	0.689	0.019	65 (11.7)	23 (8.3)	0.151	0.052	0.334	**0.034**
Alcohol use to initiate sleep ≥ 3 times per week	12 (2.2)	3 (1.1)	0.408	0.038	16 (2.9)	13 (4.7)	0.228	0.046	0.234	**0.012**

*IR, insulin resistance; p-value of probability for comparison between groups (p < 0.05 in bold); SD, standard deviation*.

### Logistic Regression Analyses of Sleep Complaints After the 10-Year Follow-Up

The results of the logistic regression analyses of different sleep complaints are presented in Table 3. Logistic regression analysis showed that IR was significantly associated with increased frequency of the following sleep complaints: sleep latency (reflecting difficulty to fall asleep) [odds ratio (OR) 1.40, 95% CI 1.04–1.89; *p* = 0.027], early waking up in the morning (OR 1.33, 95% CI 1.01–1.74; *p* = 0.041), snoring (OR 1.87, 95% CI 1.48–2.35; *p* < 0.001), very loud snoring (OR 1.61, 95% CI 1.11–2.35; *p* = 0.013), and breathing pauses during sleep (OR 1.82, 95% CI 1.11–2.99; *p* = 0.018).

**Table 3:** Logistic regression between insulin resistance and sleep complaints.

**Independent variable**	**Model 1 OR (95% CI)**		***p***	**Model 2 OR (95% CI)**		***p***	**Sensitivity, %**	**Specificity, %**
**Difficulties falling asleep**								
IR without (1)/with (2)	1.14	(0.85–1.53)	0.380	1.06	(0.74–1.49)	0.738	14.4	87.2
**Sleep latency** **>30 min**.								
IR without (1)/with (2)	1.40	(1.04–1.89)	**0.027**	1.37	(1.01–1.93)	**0.043**	14.9	88.9
**Difficulties maintaining sleep**								
IR without (1)/with (2)	1.16	(0.95–1.43)	0.146	1.03	(0.81–1.30)	0.842	48.6	55.2
**Awakenings**								
IR without (1)/with (2)	1.17	(0.88–1.54)	0.280	1.04	(0.75–1.43)	0.822	16.4	85.6
**Awakenings too early in the morning**								
IR without (1)/with (2)	1.33	(1.01–1.74)	**0.041**	1.28	(0.94–1.75)	0.118	18.5	85.4
**Poor self-perceived sleep**								
IR without (1)/with (2)	1.26	(0.90–1.77)	0.173	1.21	(0.82–1.78)	0.338	11.0	91.1
**Excessive sleepiness in the morning**								
IR without (1)/with (2)	0.93	(0.69–1.25)	0.631	0.99	(0.70–1.40)	0.957	13.7	85.5
**Excessive sleepiness in the day**								
IR without (1)/with (2)	0.84	(0.61–1.15)	0.273	0.81	(0.56–1.17)	0.266	11.0	87.2
**Sleeping time** **≤6**								
IR without (1)/with (2)	1.22	(0.95–1.55)	0.116	1.29	(0.98–1.70)	0.070	23.9	79.4
**Napping during the daytime**								
IR without (1)/with (2)	1.13	(0.82–1.55)	0.466	0.91	(0.63–1.32)	0.625	11.9	89.3
**Snoring**								
IR without (1)/with (2)	1.87	(1.48–2.35)	**<0.001**	1.37	(1.05–1.79)	**0.020**	32.2	79.7
**Very loud snoring**								
IR without (1)/with (2)	1.61	(1.11–2.35)	**0.013**	1.34	(1.04–1.74)	**0.026**	9.4	94.0
**Breathing pauses**								
IR without (1)/with (2)	1.82	(1.11–2.99)	**0.018**	1.13	(0.63–2.02)	0.693	5.6	96.9
**Inability to work due to disturbed sleep**								
IR without (1)/with (2)	1.10	(0.793–1.52)	0.568	1.17	(0.61–2.25)	0.628	3.8	97.2
**Regular use of sleeping pills**								
IR without (1)/with (2)	0.81	(0.54–1.22)	0.314	0.71	(0.44–1.14)	0.156	6.1	92.5
**Alcohol use to initiate sleep**								
IR without (1)/with (2)	1.15	(0.62–2.14)	0.436	1.22	(0.61–2.46)	0.576	2.9	97.5

*OR, odds ratio; CI, confidence interval; IR, insulin resistance; p <0.05 in bold.*

*Model 1, insulin resistance; Model 2, insulin resistance, adjusted for a 10-year period, sex, age, body mass index, physical activity, education, systolic and diastolic blood pressures, disease, total cholesterol, triglyceride, metabolic syndrome, diabetes mellitus*.

After adjusting for the 10-year period, sex, age, physical activity, education, systolic and diastolic blood pressures, presence of disease, total cholesterol, triglyceride, MetS and DM (Model 2), IR was statistically significantly more frequent in subjects with increased sleep latency (OR 1.37, 95% CI 1.01–1.93; *p* = 0.043). These complaints are symptoms of disturbed sleep due to insomnia. Other significant changes were observed in complaints characterizing breathing disturbances during sleep. There was a statistically significant relationship between IR and increase in snoring (OR 1.37, 95% CI 1.05–1.79; *p* = 0.020) and very loud snoring (OR 1.34, 95% CI 1.04–1.74, *p* = 0.026), but not between IR and breathing pauses (OR 1.13, 95 % CI 0.63–2.02; *p* = 0.693).

### Logistic Regression Analyses of Cardiovascular Risk Factors After the 10-Year Follow-Up

The results from the logistic regression analyses between IR and cardiovascular risk factors are presented in Table 4. In subjects with IR, after adjusting for the 10-year period, age, sex and education (Model 2), logistic regression analysis showed that after 10 years, there was a significantly higher chance of deteriorating cardiovascular risk factors: cardiovascular diseases (OR 1.55, 95% CI 1.14–2.10 *p* = 0.005), hypertriglyceridemia (OR = 2.75, 95% CI 2.08–3.64, *p* < 0.001), MetS (OR = 6.75, 95% CI 5.28–8.63 *p* < 0.001), DM (OR = 6.15, 95% CI 4.05–9.35, *p* < 0.001) and obesity (OR = 5.90, 95% 4.67–7.47 CI, *p* < 0.001).

**Table 4:** Logistic regression between insulin resistance and cardiovascular risk factors.

**Independent variable**	**Model 1 OR (95% CI)**		***p***	**Model 2 OR (95% CI)**		***p***	**Sensitivity, %**	**Specificity, %**
**Cardiovascular disease**
IR without (1)/with (2)	1.83	(0.38–2.42)	**<0.001**	1.55	(1.14–2.10)	**0.005**	86.7	21.9
**Low physical activity**
IR without (1)/with (2)	1.13	(0.91–1.40)	0.286	1.12	(0.90–1.40)	0.304	33.1	69.5
**Hypercholesterolemia**
IR without (1)/with (2)	1.50	(1.19–1.88)	**<0.001**	1.19	(0.94–1.50)	0.145	75.1	35.1
**Hypertriglyceridemia**
IR without (1)/with (2)	2.79	(2.12–3.68)	**<0.001**	2.75	(2.08–3.64)	**<0.001**	23.8	89.9
**Metabolic syndrome**
IR without (1)/with (2)	6.56	(5.17–8.34)	**<0.001**	6.75	(5.28–8.63)	**<0.001**	51.4	89.0
**Smoking regular**
IR without (1)/with (2)	1.15	(0.89–1.49)	0.295	1.15	(0.87–1.52)	0.333	13.7	84.7
**Diabetes mellitus**
IR without (1)/with (2)	6.14	(4.08–9.24)	**<0.001**	6.15	(4.05–9.35)	**<0.001**	16.2	96.9
**Obesity**
IR without (1)/with (2)	5.68	(4.50–7.14)	**<0.001**	5.90	(4.67–7.47)	**<0.001**	58.3	82.2

*OR, odds ratio; CI, confidence interval; IR, insulin resistance; p < 0.05 in bold.*

*Model 1, insulin resistance; Model 2, insulin resistance, adjusted for a 10-year period, sex, age, education*.

## Discussion

Our results showing an increased number of people with BMI ≥ 30 in the IR group are in parallel with literature data demonstrating the relationship between obesity and the development of DM and cardiovascular diseases (6, 31). We found that the major cardiovascular risk factors such as arterial blood pressure, obesity and triglyceridemia were more frequently observed in the IR group, and were significantly increased after a 10-year period. These factors may be responsible for the higher incidence of cardiovascular diseases in the IR group (16, 32). A study of non-diabetic subjects, recruited from a cross-sectional population-based study in Gran Canaria Island, Spain also demonstrated that subjects with impaired glucose tolerance had IR and more cardiovascular risk factors (33). Experimental and clinical studies clearly demonstrate that increased glucose levels and impaired insulin signaling are potent drivers of the atherosclerotic process, even in the absence of concomitant risk factors such as hypertension, obesity, and dyslipidaemia (34). In line with our findings, the population-based study by Wang at al. (35) has previously shown that diastolic blood pressure decreases with age after 60 years of age.

The literature data about sleep disturbances and IR show that poor sleep has a negative impact on glucose metabolism, obesity, development of DM and arterial hypertension (6). A cross-sectional association between short sleep duration (generally <6 h per night) and increased BMI or obesity, prevalence of diabetes and hypertension and markers of cardiovascular disease has been observed in multiple studies (6). Shorter (<6 h) and longer (>9 h) durations of sleep have been adversely related to IR (27). We found that the IR group displayed more cases of longer sleep latency and early awakening in the morning after the 10-year period, the characteristic symptoms of insomnia.

Our data demonstrating an increase in sleep complaints after the 10-year period in the IR group is in accordance with laboratory and epidemiological data reporting that insufficient sleep has been linked to reduce insulin sensitivity and increased risk of type 2 DM (16, 32). If sleep is disturbed toofrequently, the sympathetic tone is elevated and it results in a higher load on the circulatory system, a higher rate of basal metabolism, a higher level of stress hormones, and, finally, a higher risk of developing IR or DM. On the other hand, DM itself, when accompanied with poor metabolic control is mostly followed by sleep disturbances. Disturbed sleep appears to modulate the risk of cardiovascular diseases and mortality associated with MetS. Fernandez-Mendoza J. and colleagues found that the hazard ratio of all-cause and cardiovascular disease/cerebrovascular mortality associated with MetS was higher for individuals who slept <6 h, as compared with those who slept >6 h; phenomenon may be linked to greater central autonomic and metabolic dysfunction in short-sleeping individuals (36).

Sleep duration may increase triglycerides and HDL levels, both of which are components of MetS. A study of Japanese people discovered that among women, both short and long sleep durations are associated with a high serum triglyceride levels or low HDL cholesterol levels, while in men, the relative risk of a high LDL -cholesterol level was lower (37). Another study of Dutch adults found a clear association between short sleep duration and elevated BMI and obesity; while levels of total cholesterol, HDL -cholesterol, triglycerides and blood pressure were associated with sleep duration (38).

The relationship between disturbed sleep and DM is two-sided, as chronic sleep disturbances elevates the risk of developing IR, while diabetes worsens the quality of sleep. Sleep disturbances significantly increase the risk of developing diabetes and cardiovascular diseases (6, 31). Disturbed sleep leads to the development of IR and beta-cell dysfunction through various pathways: hypoxia, sleep fragmentations, and the activation of the sympathetic nervous system (39). Sleep fragmentation results in elevated sympathetic activity and a higher level of inflammation (40).

Investigation of the association between sleep duration, obesity, adipokines and IR in 2,848 participants including 593 with inflammatory marker data showing high risk of type 2 DM, showed an independent relationship between long sleep duration and IR (41). Fasting insulin levels and LAR (leptin: adiponectin ratio—a measure of whole-body insulin sensitivity) were positively associated with sleep duration, while adiponectin level were negatively associated. Short and long sleep duration were independently and significantly associated with higher BMI, body weight and waist circumference (41).

We have found an interesting result that sleep complaints with disturbed breathing were characteristic of individuals with IR. Loud snoring interrupted by breathing pauses is a serious symptom of sleep apnoea and it is significantly associated with IR. This is confirmed by Bonsignore and colleagues (42) who demonstrated the association between IR, excessive daytime sleepiness and MetS in obstructive sleep apnoea patients. They also found that the number of MetS components correlated with the HOMA index. Our data agree with the Namwon Study of 10,667 healthy individuals free of DM, which demonstrated a strong association between snoring and hemoglobin A1c (HbA1c) level among females but not males (43). However, the Korean Health and Genome Study on non-obese normoglycemic adults found that snoring is significantly associated with elevated HbA1c levels in both males and premenopausal females (44). These reports support our data suggesting that snoring and breathing pauses, which are serious sleep apnoea symptoms, are associated with increased IR. On the other hand, sleep apnoea is newly established risk factor not only for arterial hypertension, but also for coronary artery disease and myocardial infarction (45). This means that snoring is as serious sleep complaint associated with increased IR and cardiovascular diseases.

Disturbed sleep may lead to obesity, IR, and development of DM, as well as to development of cardiovascular risk factors and cardiovascular diseases. Because of this, the use of simple, low-cost methods for the diagnostics of disturbed sleep and the management of sleep complaints may in many cases significantly contribute to the prevention of cardiovascular diseases and DM.

The present study has some strengths and limitations to be addressed. The strengths of our study include a reasonable sample size that allowed us to adjust our findings for variables that could potentially contribute significantly to our understanding of IR in relation to cardiovascular risk factors and sleep complaints in humans. Another strength of the current study is the large number of biomarkers measured. A great strength of this epidemiologic longitudinal cohort study was its ability to draw attention to IR, as this condition remains an important public health issue that can accompany a variety of risk factors. The main limitation of the present study is that blood samples of insulin were obtained from only in the second survey in 2013, which prevented us from evaluating the presence of IR at both timepoints.

## Conclusions

Our results demonstrate that IR is associated with the development of major cardiovascular risk factors and sleep complaints. The incidence of obesity, MetS, DM, elevated fasting glucose level, triglyceridemia and sleep complaints was more frequent after a 10-year period in subjects with IR. After a 10-year period, IR was significantly associated with an increase in sleep complaints, including sleep latency reflecting difficulty in falling asleep, snoring and very loud snoring.

## Data Availability Statement

The raw data supporting the conclusions of this article will be made available by the authors, without undue reservation.

## Ethics Statement

The studies involving human participants were reviewed and approved by Bioethics Committee of Lithuania (Protocol Code BE-2-25, Approved 14 June 2012). The patients/participants provided their written informed consent to participate in this study.

## Author Contributions

NK: conceptualization and formal analysis. AP, GV, and NM: methodology. NK, AP, GV, and NM: validation. GV and NM: investigation, writing—review and editing, and supervision. AP: data curation. NK and AP: writing—original draft preparation. NM: lead of the project. All authors have read and agreed to the published version of the manuscript.

## Funding

This research was funded by a Grant (No. S-SEN-20-13) from the Research Council of Lithuania.

## Conflict of Interest

The authors declare that the research was conducted in the absence of any commercial or financial relationships that could be construed as a potential conflict of interest.

## Publisher's Note

All claims expressed in this article are solely those of the authors and do not necessarily represent those of their affiliated organizations, or those of the publisher, the editors and the reviewers. Any product that may be evaluated in this article, or claim that may be made by its manufacturer, is not guaranteed or endorsed by the publisher.
